# Effects of water recirculation rate on the microbial community and water quality in relation to the growth and survival of white shrimp (*Litopenaeus vannamei*)

**DOI:** 10.1186/s12866-019-1564-x

**Published:** 2019-08-19

**Authors:** Zhao Chen, Zhiqiang Chang, Long Zhang, Yuli Jiang, Hongxing Ge, Xiefa Song, Shibo Chen, Fazhen Zhao, Jian Li

**Affiliations:** 1 Key Laboratory for Sustainable Development of Marine Fisheries, Ministry of Agriculture, Yellow Sea Fisheries Research Institute, Chinese Academy of Fishery Sciences, Qingdao, 266071 People’s Republic of China; 2 Fisheries College, Ocean University of China, Qingdao, 266003 People’s Republic of China; 3 Qingdao Excellent Ocean Group Co., Ltd, Qingdao, 266400 People’s Republic of China

**Keywords:** RAS, Recirculation ratio, HRT, *Litopenaeus vannamei*, Biofilm, Bacterial community

## Abstract

**Background:**

Microbial community and its management are crucial to the stabilization of culture environment for recirculating aquaculture system (RAS). Although several studies have been carried out for the microbial community of RAS, few studies were on the RAS for shrimp. Water recirculation ratio is an important factor for the microbial community and the management of RAS. Therefore, low (LC), medium (MC) and high (HC) recirculation ratio systems were set to explore the microbial community constitution of RAS for *Litopenaeus vannamei* and study the effect of water recirculation rate on it.

**Results:**

The bacterial community of bioreactor was mainly dominated by *Proteobacteria* (41.6–70.7%), followed with *Planctomycetes* (12.5–31.0%), *Bacteroidetes* (10.5–26.0%), *Actinobacteria* (1.1–4.8%) and *Verrucomicrobia* (1.4–6.8%) phylum. The most dominant family of bioreactor was *Rhodobacteraceae* or *Planctomycetaceae*. The bacterial community of culture water was simpler than bioreactor and dominated by *Proteobacteria* (61.8–96.4%). The dominant bacterial groups of bioreactor and culture water are also different among the three water recirculation rates, and the proportions of dominant groups showed a trend with the variety of water recirculation rate. Water quality indexes including ammonia and nitrite decreased with the increasing of water recirculation rate. According to the growth performance of *L. vannamei*, shrimp had better performance of growth rate and final weight in MC and HC, however, shrimp had higher survival and yield in LC. Shrimp survival and yield had an inverse correlation with water recirculation rate.

**Conclusions:**

The results demonstrate the microbial community of RAS for shrimp, highlight the importance of further studies on the function of bacterial taxa, and promote the understanding of the effects of water recirculation rate on the microbiota. The findings suggest that water recirculation rate has important impacts on the microbial community, water quality and shrimp growth. Increasing the water recirculation rate could improve the water quality and promote the growth of shrimp. However, the survival rate and yield of *L. vannamei* are higher under low water recirculation rate. Recirculation rate is an effective method to manage RAS, and its impact on RAS needs further study, especially in the application of low level of water recirculation.

## Background

In response to increasing demand for aquaculture products and a strict requirement on aquaculture wastewater discharge, land-based recirculating aquaculture system (RAS) is being developed as a viable eco-sustainable alternative to traditional aquaculture because of its minimal environmental impact and controlled culture condition [1]. After a long period of development, the technology of RAS gradually matures in fish culture. RAS is also feasible and available for shrimp culture [2–5] whereas its application is relatively scarce in shrimp culture. The shrimp farming industry is an important component of world aquaculture [6]. Nevertheless, shrimp farming also suffered from many problems as traditional aquaculture, including disease outbreaks, environmental degradation, and poor management [5, 7–9]. The Pacific white shrimp (*Litopenaeus vanname*) is one of the major shrimp farming species and is adaptive to intensive farming for its strong adaptability. RAS is a well alternative mode for *L. vanname* culture.

The microorganism is crucial to the stability of the aquaculture environment in RAS. The biofilter is a microbial purification link of RAS and acts to remove nitrogenous waste byproducts generated by fish protein catabolism and oxidation processes [10]. However, biofilter is a complicated black box, and the understanding of its action mechanism is incomplete. As for the efficiency of the biofilter, the primary determinants are the communities and abundances of the functional microbial groups established within the filter, which determine the final results [11]. Microorganisms in culture water are also important for the stability of the aquaculture environment and the health of aquaculture organisms since they could directly contact with culture organisms. Therefore, information on the bacterial community and diversity in biofilters and culture water would be useful for the design and operation of RAS [12, 13].

The understanding of the internal structure of biofilters is gradually increased with many studies about the microbial community in biofilters and culture water have been done [13–20]. Various factors could affect the microbial community [14, 17], which include culture species and hydraulic retention time (HRT). HRT directly related to water recirculation ratio or flow rate and is an important factor for microbial community structure [14, 21–23] and biofilters efficiency [24, 25]. However, most studies of HRT was merely about reactors rather than based on aquaculture system, much less about RAS of shrimp. To maintain the well aquatic environment RAS adopt high water recirculation ratio (more than 20 cycles per day) [26–29], since high recirculation ratio is in beneficial to remove nitrite, ammonia and solid particle [24, 25]. However, high water recirculation ratio means high energy consumption. Besides, *L. vanname* has a stronger tolerance to nitrite and ammonia [30, 31] than fish and could be cultured in turbid water with the mass of suspended particles. Therefore, RAS of shrimp could adopt low water recirculation ratio for energy conservation. It’s necessary to carry out studies on the effects of water recirculation ratio on the microbial community for improving the management of microorganism in the recirculating shrimp culture system.

The objective of the present study was to characterize the bacterial community in the water and on the filters of biofilters, and explore the effects of water recirculation rate on the microbial community, water quality and shrimp, and determine the relationship of microbial community, water quality with shrimp growth and survival. And thus, improving the management of RAS for *L. vannamei*.

## Results

### Microbial community composition of the bioreactor

**Fig. 1:**
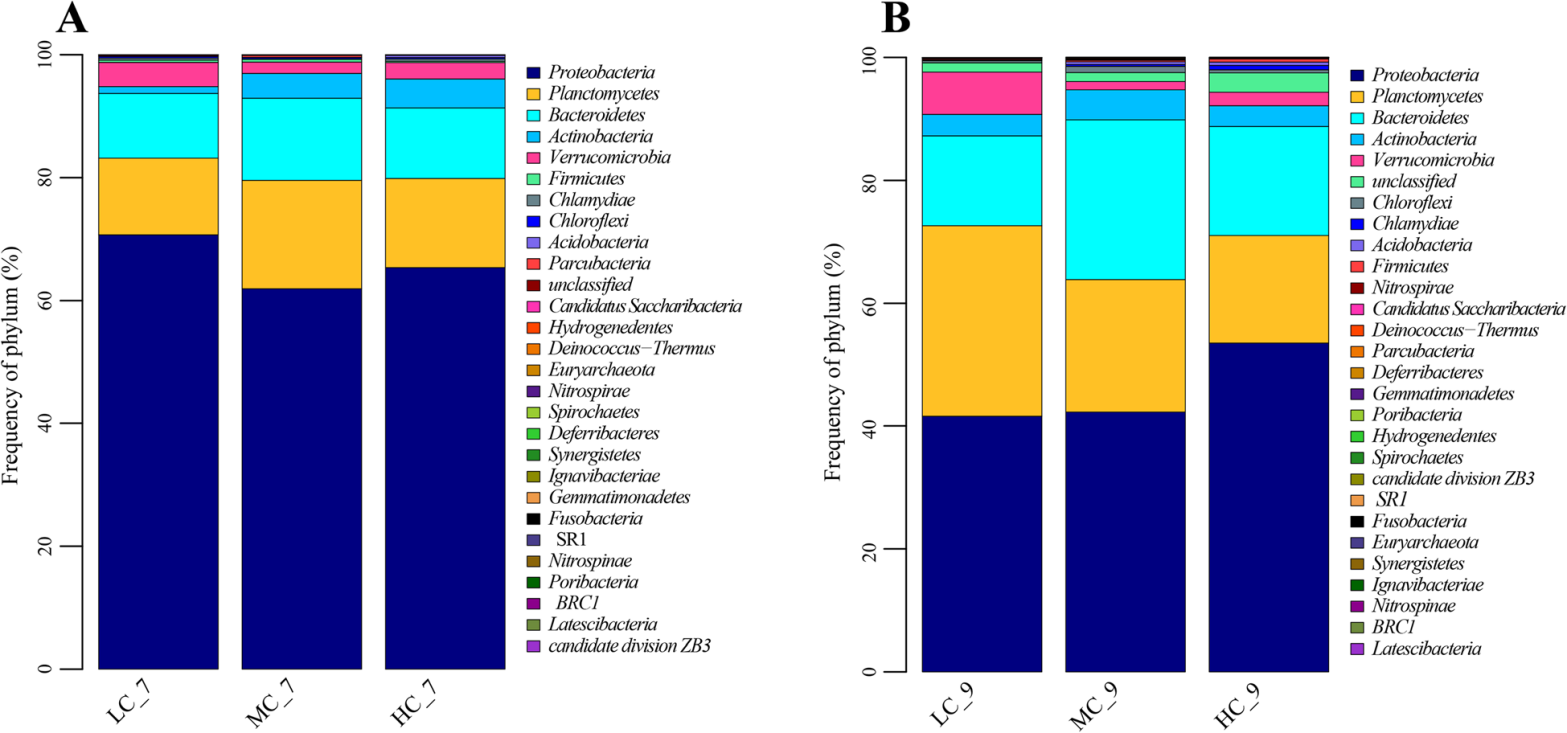
Taxonomic assignment of bacteria in the bioreactor at phylum level shown as a percentage of the total microbiota. **a** and **b** are the microbial community structure in midterm (July) and terminal (September) of the experiment respectively

The bacterial community in the bioreactor was mainly composed of *Proteobacteria* (41.6–70.7%), *Planctomycetes* (12.5–31.0%), *Bacteroidetes* (10.5–26.0%), *Actinobacteria* (1.1–4.8%) and *Verrucomicrobia* (1.4–6.8%) phylum (Fig. 1), which account for probably 98% of the total bacteria in frequency. *Proteobacteria* dominated all bioreactor communities in the experiment.

**Fig. 2:**
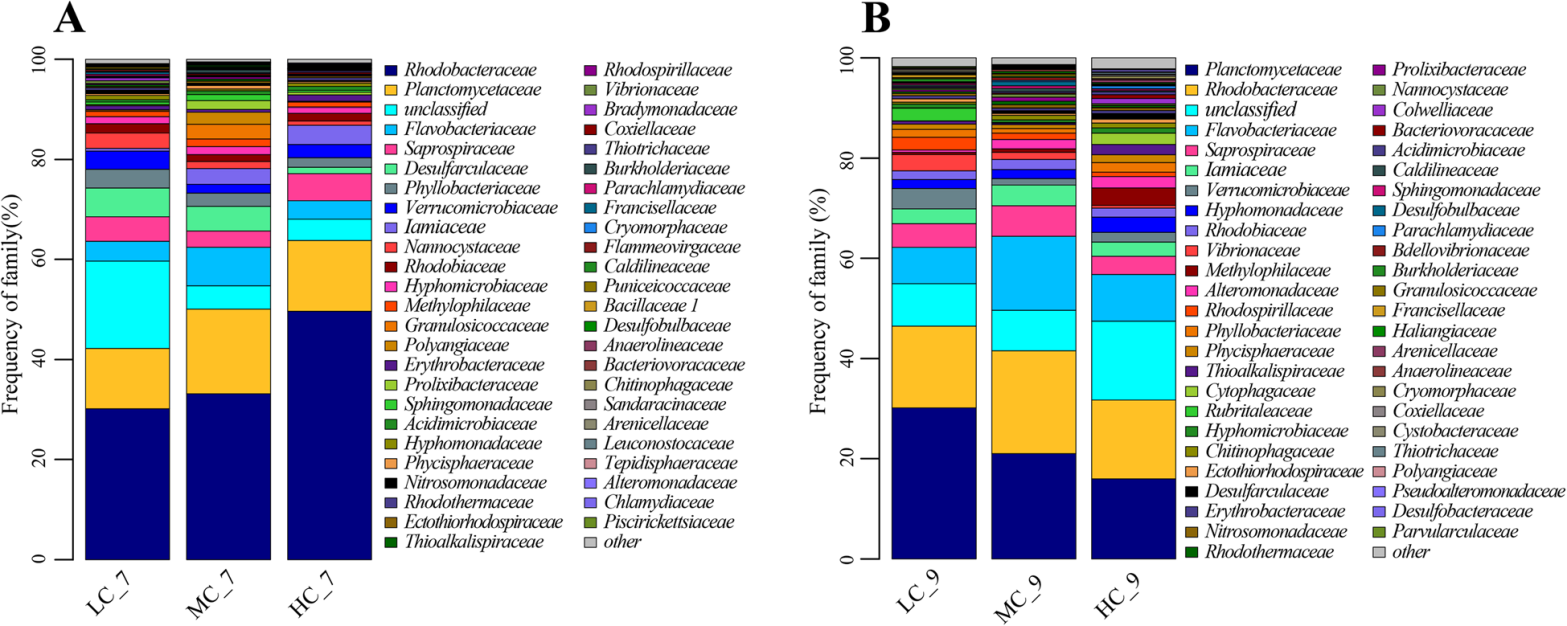
Taxonomic assignment of bacteria in the bioreactor at the family level shown as a percentage of the total microbiota. **a** and **b** are the microbial community structure in the midterm (July) and terminal (September) of experiment respectively

Microbial community composition changed with time. At the phylum level, the percentage of *Proteobacteria* reduced from 61.9–70.7% to 41.6–53.5% (Fig. 1a, Fig. 1b). At the family level, the dominant bacteria group turned from *Rhodobacteraceae* (30.2–49.6%) to *Planctomycetaceae* (16.01–30.14%) (Fig. 2a, Fig. 2b). Besides, the frequency of dominant families presented a trend with water recirculation rate and was significantly affected by it (*P* < 0.05).

**Fig. 3:**
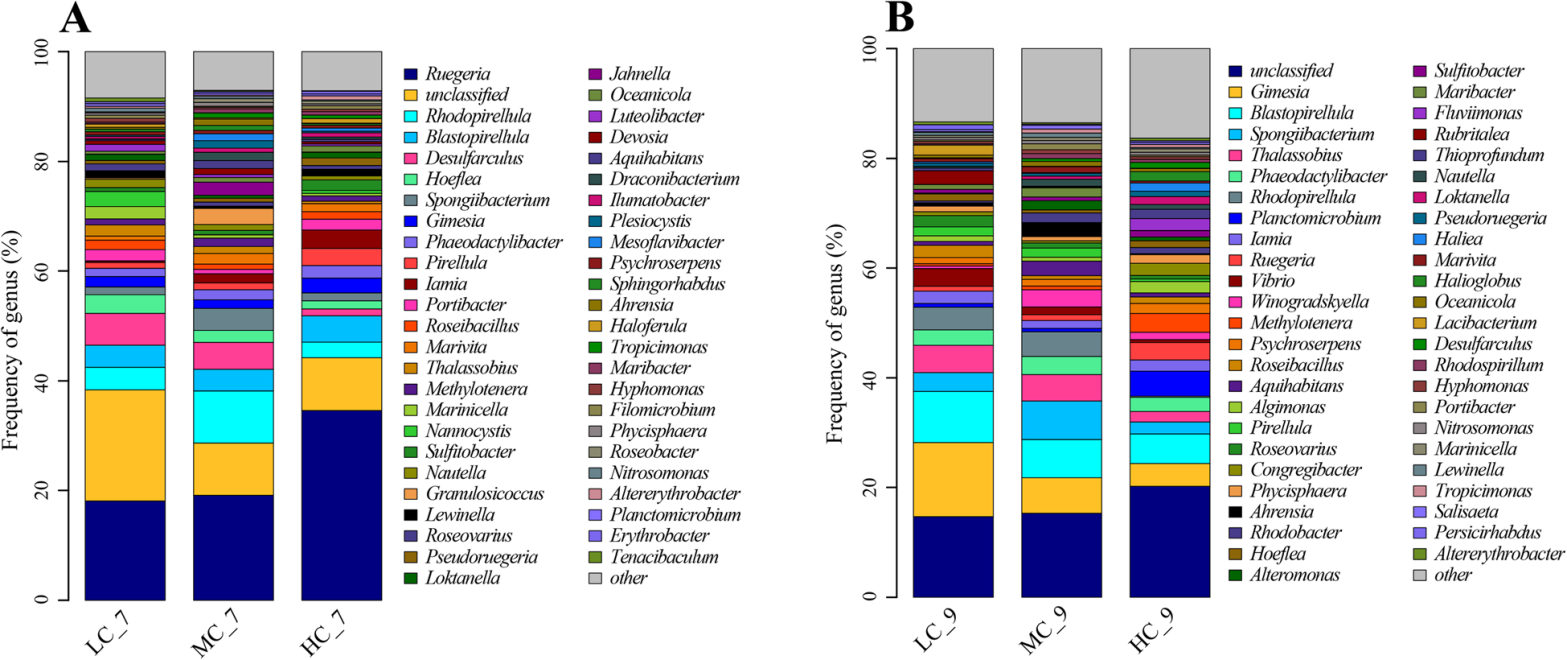
Taxonomic assignment of bacteria in the bioreactor at genus level shown as a percentage of the total microbiota. **a** and **b** are the microbial community structure in the midterm (July) and terminal (September) of experiment respectively

At the genus level, 30–50% of the bacteria belong to unclassified or other bacteria whose abundance was very low. In the midterm (Fig. 3a), the most dominant genus was *Ruegeria*, accounting for 18.2–34.6% of total bacteria. *Nitrosomonas* was the only nitrobacteria detected in all bioreactors with the frequency of 0.2–1.0%. At the end of culture (Fig. 3b), the *Ruegeria* was not the dominant genus anymore. And the dominant genus was different in LC, MC and HC. In LC, the most dominant genus was *Gimesia* (13.5%), while in MC, the dominant genus including *Spongiibacterium* (7.0%), *Blastopirellula* (7.0%) and *Gimesia* (6.5%), and in HC, the dominant genus including *Blastopirellula* (5.4%), *Planctomicrobium* (4.6%) and *Gimesia* (4.2%). The proportion of dominant genus was declined with the rise of water recirculation. Three kinds of nitrobacteria were detected in the end including *Nitrococcus* (0.0–0.6%), *Nitrosomonas* (0.2–0.8%) and *Nitrospira* (0.0–0.3%). The proportion of nitrobacteria, in the end, was more than that in the midterm. *Vibrio* as potential pathogeny whose percentage was increased from 0.1–1.1% in the midterm to 0.5–3.6% in the end. And the proportions of *Vibrio* in bioreactors were declined with the rise of water recirculation (*P* < 0.05) in the end.

### Microbial community composition of culture water

**Fig. 4:**
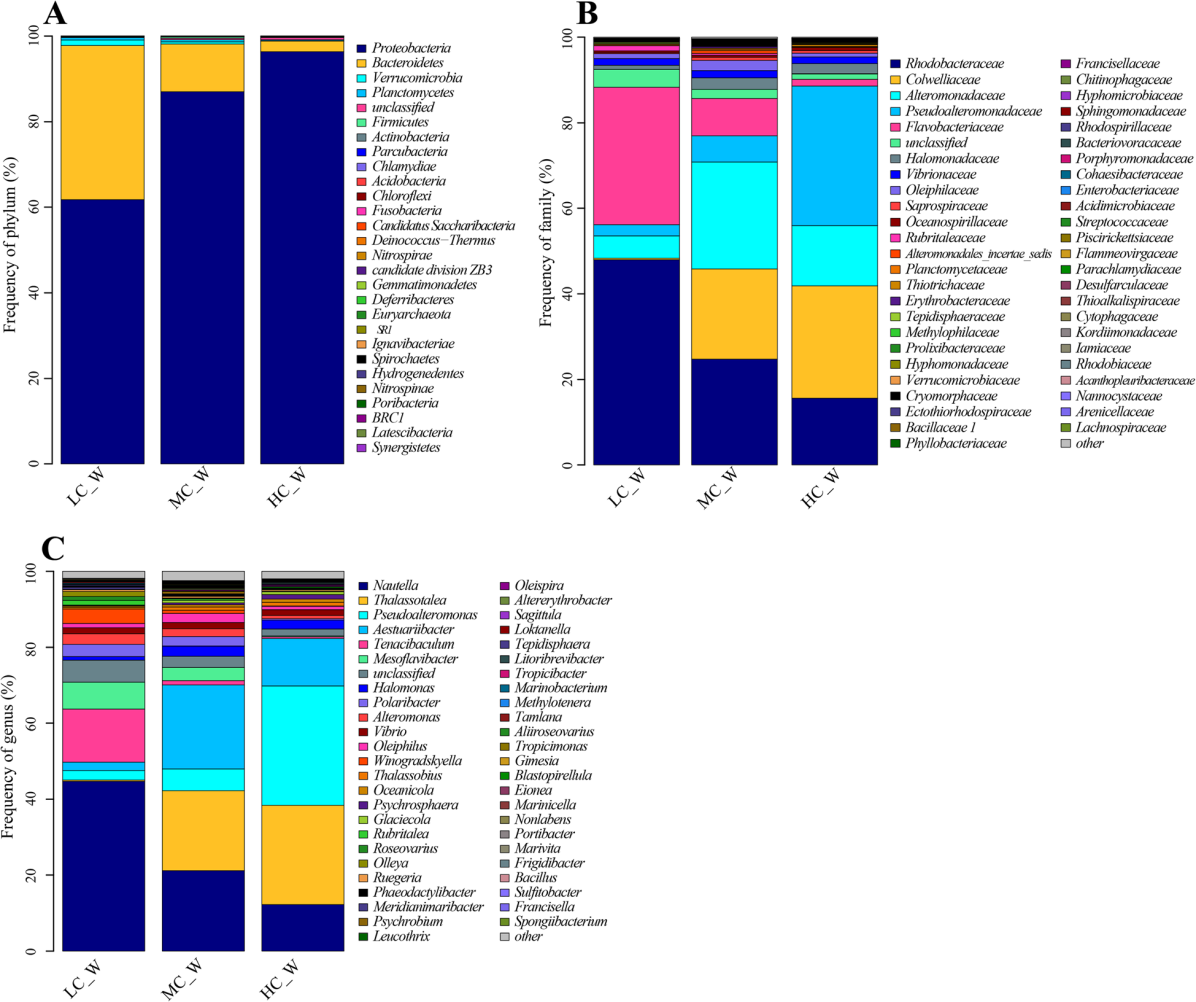
Taxonomic assignment of bacteria in culture water at phylum (**a**), family (**b**) and genus (**c**) level shown as a percentage of the total microbiota in the terminal (September) of experiment respectively

As shown in Fig. 4, the bacteria composition of culture water was simpler than that of the bioreactor. At the phylum level, the bacteria community was mainly composed of *Proteobacteria* and *Bacteroidetes*, which roughly accounted for 98% of the total bacteria. Meanwhile, both the proportion of *Proteobacteria* and *Bacteroidetes* was affected by water recirculation (*P* < 0.05). Furthermore, at family and genus level, all proportions of dominant groups presented a trend with water recirculation rate.

At the family level, the bacteria community was mainly dominated by *Rhodobacteraceae* (15.6–48.0%), *Colwelliaceae* (0.4–26.2%), *Alteromonadaceae* (5.2–25.0%), *Pseudoalteromonadaceae* (2.6–32.6%) and *Flavobacteriaceae* (1.6–32.1%). The five families roughly accounted for 90% of the total bacteria. The frequencies of *Rhodobacteraceae* and *Flavobacteriaceae* were declined with the rise of water recirculation. However, the frequencies of *Colwelliaceae* and *Pseudoalteromonadaceae* were increased with the rise of water recirculation. And *Alteromonadaceae* had the highest frequency in MC. Besides, the proportion of *Flavobacteriaceae* was significantly affected by water recirculation (*P* < 0.05).

At the genus level, the dominant bacterial community was composed of *Nautella* (12.2–44.6%), *Thalassotalea* (0.3–26.2%), *Pseudoalteromonas* (2.5–31.4%), *Aestuariibacter* (2.2–22.2%) *and Tenacibaculum* (0.5–14.0%). The sum proportion of the five genera accounted for 62.5–82.8%. And the frequency of other portion which had very low abundance was less than 2.5%. All the frequencies of five dominant genera showed a trend impacted by water recirculation. The dominant genus was different in LC, MC and HC. In LC, the dominant genera were *Nautella* (44.6%) and *Tenacibaculum* (14.0%), while in MC, the dominant genera including *Aestuariibacter* (22.2%), *Thalassotalea* (21.1%) and *Nautella* (21.2%), and in HC, the dominant genera including *Pseudoalteromonas* (31.4%), *Thalassotalea* (26.2%), *Aestuariibacter* (12.4%) and *Nautella* (12.2%). *Nitrosomonas* and *Nitrospira* were detected in culture water. But the proportion was less than 0.1%. The frequency of potential pathogen *Vibrio* was about 1.5%.

### The diversity of the microbial community

43,805–71,225 sequences with an average of 55,410 were detected in one bioreactor sample or culture water sample (Table 1). 744–1272 OTUs with an average of 1026 could be obtained from one sample. There was no significant difference (*P* > 0.05) between bioreactor samples and culture water samples in sequence and OTU number.

**Table 1:** Alpha-diversity of bacterial community in bioreactors and water

α-diversity	LC_7	MC_7	HC_7	LC_9	MC_9	HC_9	LC_W	MC_W	HC_W
*S*_Chao1_	1388.75 ± 118.67^abc^	1307.65 ± 90.55^bcd^	1437.40 ± 160.10^abc^	1400.25 ± 151.38^abc^	1467.57 ± 87.17^abc^	1552.04 ± 22.51^ab^	1083.22 ± 78.28^d^	1600.55 ± 163.36^a^	1243.33 ± 95.83^cd^
*D*_Simpson_^*^	0.060 ± 0.042^c^	0.050 ± 0.000^c^	0.115 ± 0.106^bc^	0.020 ± 0.014c	0.020 ± 0.000^c^	0.015 ± 0.007^c^	0.220 ± 0.071^ab^	0.190 ± 0.028^ab^	0.270 ± 0.099^a^

^*^smaller values represent higher bacterial diversity

^a,b,c,d^different lowercase on superscript mean significant difference (*P* < 0.05)

**Fig. 5:**
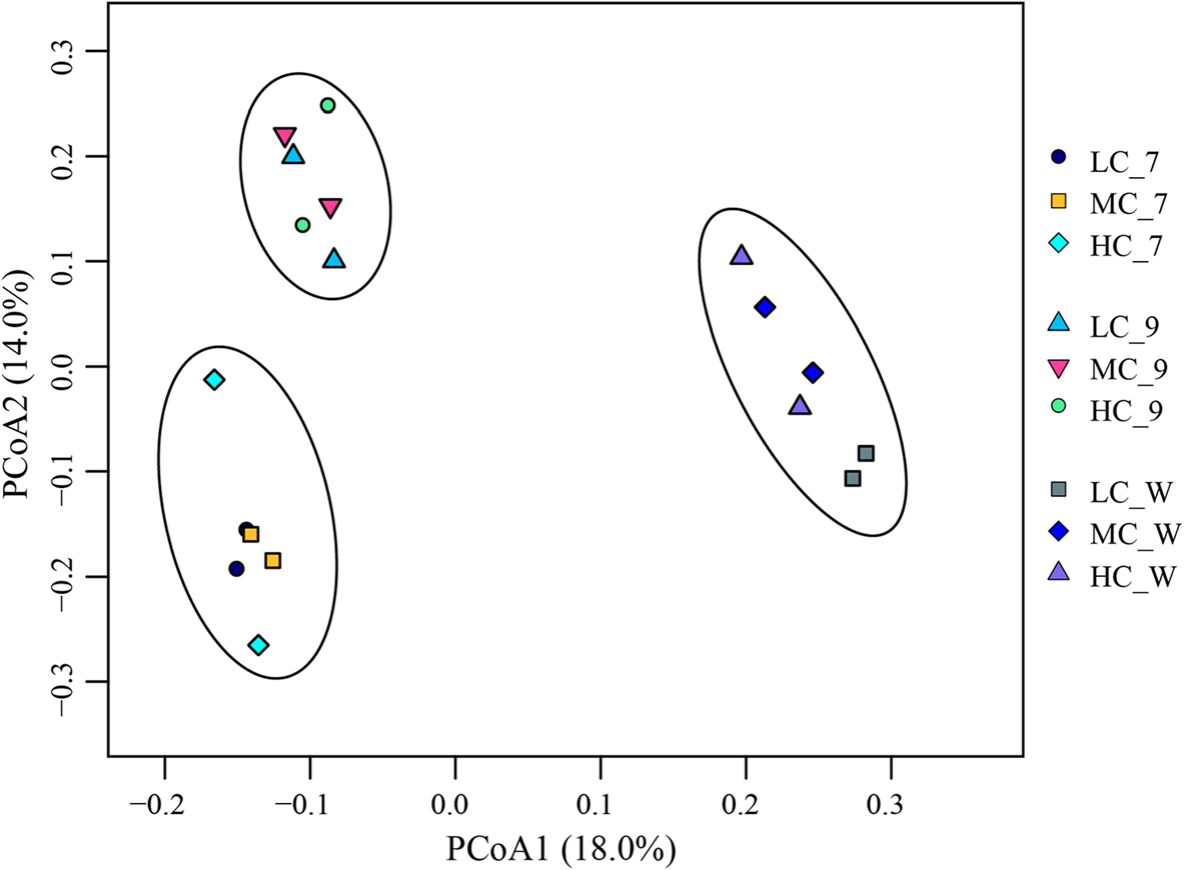
UPGMA tree of Bray-Curtis distances in the genus

**Fig. 6:**
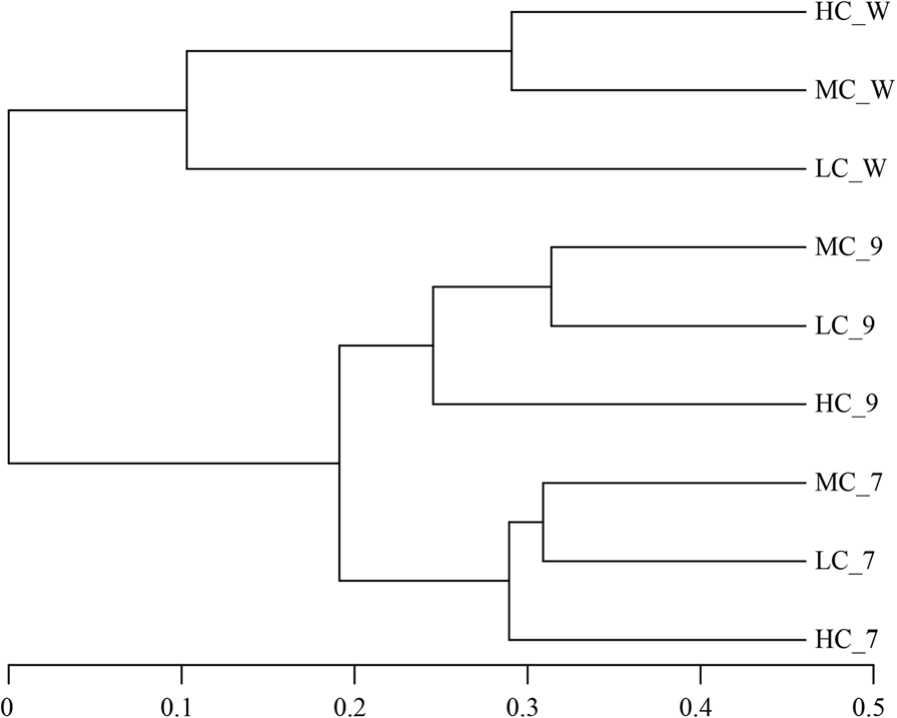
PCoA basing on the UniFrac distance of bacterial communities

According to the Chao1 index, the abundance of bacteria in bioreactors had no significant difference (*P* > 0.05) among LC, MC and HC or between midterm and terminal of the experiment. But the bacterial diversity in the terminal was higher than that in the midterm according to Simpson index. In addition, the diversity of the bacterial community in bioreactors was all higher than that in culture water. There was a significant difference of bacterial community among bioreactor samples in the midterm and end, and culture water samples in the end (Fig. 5). Besides, the difference between bioreactor samples and culture water samples was more. The water recirculation rate had a significant effect on bacterial diversity. In the midterm, bacterial diversity in HC was the lowest, however, in the end, HC had the highest bacterial diversity. According to Fig. 6, the water recirculation rate had different effects on the microbial community diversity of bioreactor and culture water. In bioreactors, LC and MC had more similar β-diversity. However, in culture water, MC and HC had more similar β-diversity.

### Water quality

As to the temperature, salinity, DO and pH during the whole experimental period, there were no significant differences (*P* > 0.05) among the three groups at different recirculation rate. However, the turbidity, vibrio count, and the concentration of ammonia and nitrite in the LC group were significantly higher (*P* < 0.05) than those groups with higher water recirculation rate (Tables 2 and 3). The peak concentration of TAN and nitrite were 1.69 and 7.32 mg L^− 1^ in LC, 0.72 and 3.87 mg L^− 1^ in MC, 0.81 and 2.81 mg L^− 1^ in HC, respectively.

**Table 2:** Initial water quality

T (°C)	Sal (g L^− 1^)	O_2_ (mg L^− 1^)	pH	Turbidity (NTU)	Vibrio (CFU mL^− 1^)	NH_4_^+^-N (mg L^− 1^)	NO_2_^—^N (mg L^− 1^)
27.85 ± 0.5	29.25 ± 0.12	6.32 ± 0.31	8.07 ± 0.03	0.00 ± 0.00	18.33 ± 7.64	0.01 ± 0.00	0.00 ± 0.00

**Table 3:** Temperature, salinity, O_2_, pH, turbidity, vibrio, ammonia and nitrite of rearing water

Treatment	T (°C)	Sal (g L^− 1^)	O_2_ (mg L^− 1^)	pH	Turbidity (NTU)	Vibrio (10^3^CFU mL^− 1^)	NH_4_^+^-N (mg L^− 1^)	NO_2_^—^N (mg L^− 1^)
LC	28.19 ± 2.35^a^	29.62 ± 0.43^a^	5.92 ± 0.30^a^	7.91 ± 0.35^a^	1.40 ± 0.53^a^	3.79 ± 3.04^a^	0.67 ± 0.46^a^	3.23 ± 2.44^a^
MC	28.27 ± 2.10^a^	29.52 ± 0.41^a^	6.12 ± 0.22^a^	8.02 ± 0.38^a^	0.81 ± 0.24^b^	1.75 ± 1.89^b^	0.39 ± 0.16^b^	2.06 ± 1.11^ab^
HC	28.31 ± 2.44^a^	29.69 ± 0.49^a^	6.03 ± 0.23^a^	8.10 ± 0.45^a^	0.67 ± 0.12^b^	2.04 ± 2.09^b^	0.28 ± 0.23^b^	1.26 ± 0.82^b^

^a, b^different lowercase on superscript mean significant difference (*P* < 0.05)

**Fig. 7:**
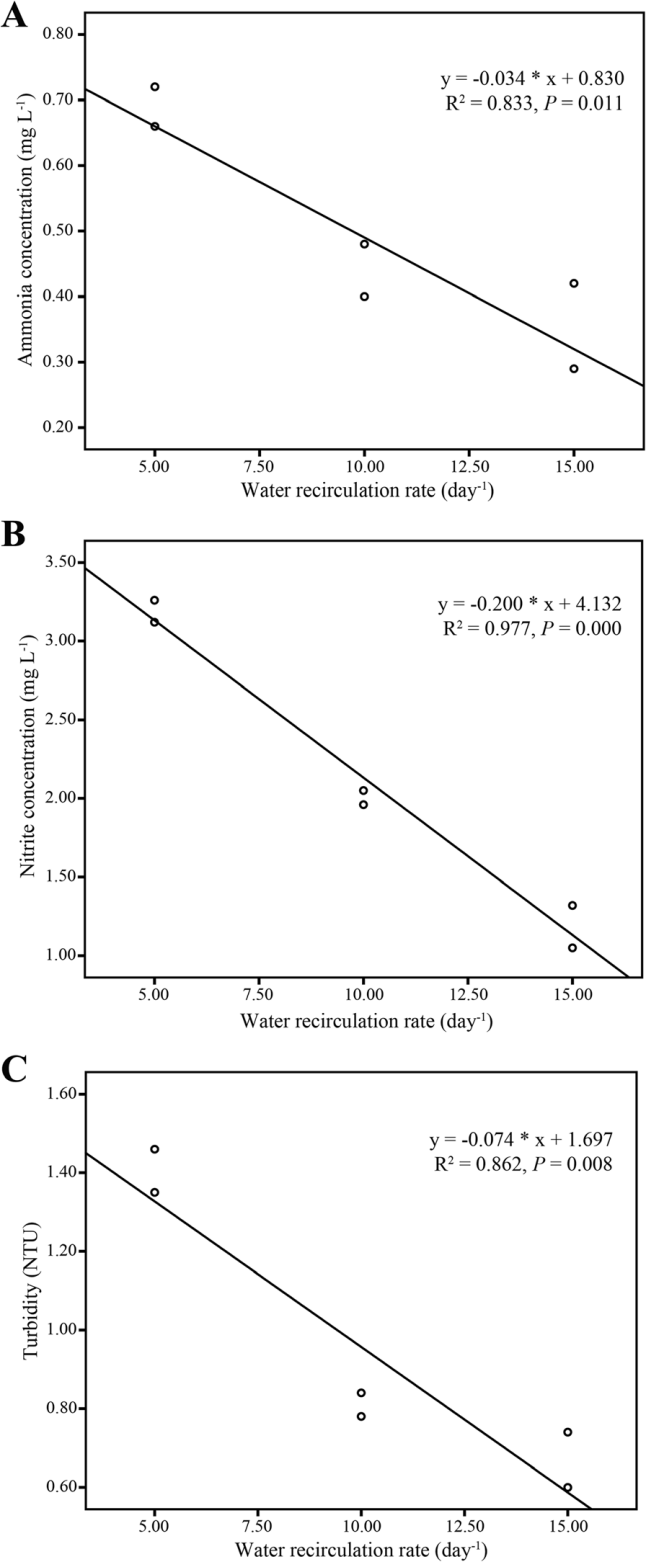
Linear regression of ammonia (**a**), nitrite (**b**) and turbidity (**c**) on amount of water recirculation ratio

The Pearson correlation analysis showed there was a significant correlation between water quality indexes of ammonia (r = −0.913, *P* < 0.05), nitrite (r = − 0.988, *P* < 0.01) and turbidity (r = − 0.928, *P* < 0.01) and water recirculation rate. Increased flow rate showed a decreasing trend in ammonia, nitrite and turbidity as shown in the regression analysis in Fig. 7. The Vibrio number also showed a decreasing trend with the increasing of water recirculation rate, but there was no significant correlation (*P* > 0.05) between them due to the great variation of Vibrio.

### Growth performance of shrimp

At harvest, significant differences were observed in final average weight, SGR, survival, and harvest yield among the three treatments (Table 4). FCR also showed some differences among the treatments, but there was no significant difference among the treatments due to the variance within the treatments.

**Table 4:** Growth performance of shrimp reared under different water recirculation ratio

Variable	Treatment		
	LC	MC	HC
Final weight (g)	6.78 ± 2.40 ^c^	9.91 ± 4.26 ^a^	8.42 ± 3.33 ^b^
SGR (%)	4.8 ± 0.4 ^b^	5.2 ± 0.5 ^a^	5.0 ± 0.4 ^a^
Survival (%)	66.2 ± 10.4 ^a^	42.5 ± 3.1 ^b^	37.9 ± 0.9 ^b^
Harvest yield (kg m^− 3^)	1.51 ± 0.33 ^a^	1.39 ± 0.05 ^b^	1.17 ± 0.17 ^b^
FCR	1.54 ± 0.34 ^a^	1.68 ± 0.01 ^a^	1.92 ± 0.23 ^a^

^a, b, c^different lowercase on superscript mean significant difference (*P* < 0.05)

**Fig. 8:**
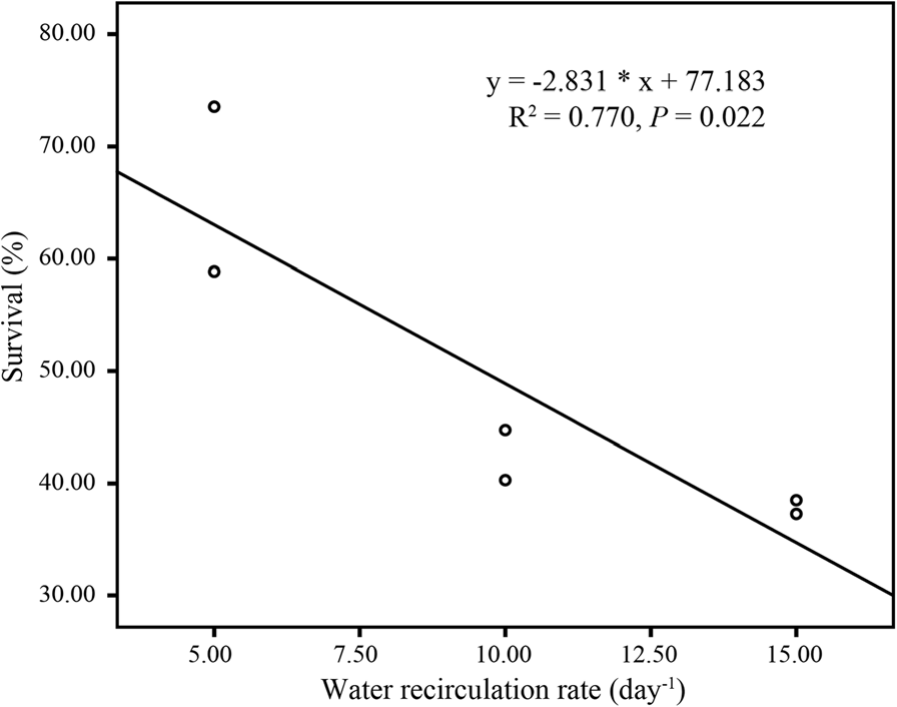
Linear regression of shrimp survival on amount of water recirculation ratio

The growth performance of shrimp was affected by water quality. The Pearson correlation analysis showed that there was a correlation between shrimp weight and ammonia concentration (*r* = − 0.676, *P* < 0.05), and between shrimp weight and Vibrio number (*r* = − 0.869, *P* < 0.01). There was no significant correlation between shrimp weight and nitrite concentration. Increasing water recirculation rate was helpful to improve water quality and promote shrimp growth. However, there was a significant inverse linear relationship (R^2^ = 0.77, *P* < 0.05) between survival and water recirculation rate (Fig. 8). The survival rate of shrimp decreased with the increase of water recirculation rate. The harvest yield and FCR also showed the inverse trend with water recirculation ratio in spite of no significant linear relationship was observed (*P* > 0.05).

## Discussion

### Microbial community structure of biofilter

The communities and abundances of the functional microbial groups are the primary determinants for aquaculture condition [11]. Previously, low-coverage characterization methods (e.g., DGGE, clone libraries) was adopted to describe the taxa present in the microbial community, but the extent of this diversity and similarity among systems was relatively unknown. Massively parallel sequencing technology is an emerging and reliable method to study the microbial community [32]. Figure 1 showed that the most dominant phylum was *Proteobacteria*, which was dominated by α-*Proteobacteria* (27.0–56.9%). Schreier [12] summarize the composition of the microbial community in recirculating aquaculture systems including *Actinobacteria*, *Bacterioidetes*/*Chlorobi*, *Firmicutes*, *Nitrospirae*, *Planctomycetales*, and *Proteobacteria*. At the phylum level, the microbial community composition has high similarity to the report of Schreier [12], as well as the research of Weitz [33] and Huang [16] in the biofilter. *Proteobacteria* is the most dominant phylum in RAS.

The taxonomic assignment of bacteria in bioreactors had many similarities to the previous reports at the phylum level. But with the decrease of classification level, the differences in the bacterial community increased gradually. Due to the limited understanding of the entire bacteria, there were 10–20% of the bacteria in bioreactors which were unclassified in the genus. And there were 20–30% of the bacteria whose proportion in the genus was lower than 1%. The most dominant genus was *Ruegeria* (19.1–34.6%) in the midterm, while in the end, the dominant genera included *Gimesia* (4.2–13.5%), *Blastopirellula* (5.4–9.4%) and *Spongiibacterium* (2.2–7.0%). The dominant genera were different from the reports of Schneider [14] and Ruan [13]. Rud fund that culture systems were dominated by *Proteobacteria* with *Rhodobacteraceae* as the dominating taxa, followed by *Bacteroidetes* that was dominated by *Polaribacter* among others [34]. Huang also reported that the microbial community composition was different among biofilters [16]. The dominant genera of this study were different from the reports above, much less the bacteria composition. A lot of factors could impact the bacterial community structure including DO [17], salinity [17], temperature [35], C/N ratio [36], cultured species [37], HRT [14], disinfection [33], etc. The microbial community in bioreactors is difficult to control [12, 38–40] and many of the inefficiencies of the system originate from this [41]. But the information on bacterial composition structure in biofilter is necessary, and the management of autotrophic and heterotrophic bacteria is the key factor to maintain good water quality and health of farmed organisms [42].

The reason why bioreactor could remove ammonia and nitrite is that of the bacteria community of biofilm. There are three validly described genera of the ammonia-oxidizing bacterium (AOB), *Nitrosomonas*, *Nitrosospira* and *Nitrosococcus*, and four genera of the nitrite-oxidizing bacterium (NOB), *Nitrobacter*, *Nitrospina*, *Nitrococcus* and *Nitrospira*, which contribute to the process of nitration. In this study, three genera of nitrobacteria were detected including *Nitrococcus*, *Nitrosomonas* and *Nitrospira*. But none of the proportion of nitrifier exceeded 1%. It was different from previous reports [13, 16]. The result of Huang showed that *Nitrospira* and *Nitrosomonas* were dominant nitrifiers in biofilters with the proportion of 0.2–16.4% and 0.1–2.8%, respectively. Ruan reported that the proportion of *Nitrospira* was 1.8–6.0%, while the proportion of *Nitrosomonas* was less than 0.1% in the bioreactor of a full-scale marine RAS. In this study, RASs was for shrimp which had a shorter culture-cycle than fish. It could be the time limit result in the low frequency of nitrobacteria since the experiment sustained only 3 months. Other bacteria which not belong to traditional nitrobacteria may participate in the nitrogen cycle, such as *Rhodobacteraceae* [43–45] and *Planctomycetaceae* [46, 47]. On the other hand, the involvement of novel nitrite-oxidizing species, which include Archaea, may contribute to nitration [12, 15, 48–50].

The function of dominant genera in bioreactors, *Ruegeria*, *Gimesia*, *Blastopirellula* and *Spongiibacterium*, is indefinite. *Ruegeria* belongs to *Rhodobacteraceae*. Lidbury reported that *Ruegeria pomeroyi* DSS-3 could contribute to the remineralization of nitrogen in the form of ammonium [45], and Choi reported *Ruegeria pomeroyi* has *nosZ* sequence for N_2_O reduction [44]. *Gimesia* genus belongs to *Planctomycetaceae* and has only one species *Gimesia maris* which was called *Planctomyces maris* [46]. *Gimesia maris* is a chemoheterotroph growing on defined medium with glucose and ammonia as sole carbon and nitrogen sources respectively [51]. *Blastopirellula* genus belongs to *Planctomycetaceae*. *Blastopirellula* is chemoheterotrophic whose type species *Blastopirellula marina* could use ammonia, nitrate and organic nitrogen [52]. *Spongiibacterium* is a genus of *Flavobacteriaceae* [53]. In consideration of the low frequency of AOB and NOB in bioreactors, *Rhodobacteraceae* and *Planctomycetaceae*, which were the dominant families of bioreactors, may contribute more to the nitrogen conversion in RAS. The function of *Rhodobacteraceae* and *Planctomycetaceae* in aquaculture need further investigation.

### Microbial community structure of culture water

Bacteria is an essential part of the breeding water which could direct contact with culture organism with lots of effects. Zheng reported that *Proteobacteria* and *Bacteroidetes* were widely distributed in healthy shrimp at all growth stages, but varied in relative abundance among different steps [54]. In this study, the composition of bacteria in culture water was mainly composed of *Proteobacteria* (61.8–96.4%) and *Bacteroidetes* (2.5–36.0%). However, at the genus level, the bacteria composition had a lot of difference from previously reports [54]. *Nautella* is a genus of *Rhodobacteraceae*. It was fund by Vandecandelaere, and has a great similarity to *Roseobacter* [55]. According to Zheng’s study, *Nautella* is pathogen to *L. vannamei* for it had more proportion in the diseased water sample and could be an indicator for monitoring the health status of shrimp larvae in the hatchery [54, 56]. The genus *Thalassotalea* was proposed by Zhang [57] with the description of *Thalassotalea piscium* as its type species and reclassification of four species of the genus *Thalassomonas* as members of the novel genus, and its description was amended by Park [58]. Hou isolated *Thalassotalea marina* sp. nov. from RAS of *Epinephelus awoara* [59]. But the relationship between *Thalassotalea* and the cultured organism is still unknown. *Pseudoalteromonas* is probiotics with the effects of reducing the larval mortality of fish and shrimp [60, 61], inhibiting vibrio [62–64] and promoting digestion [65, 66]. The genus *Aestuariibacter*, which belongs to the family *Alteromonadaceae*, was proposed by Yi for strictly aerobic, chemoheterotrophic, salt-requiring and nitrate-reducing [67]. Its effect on shrimp is still unknown. *Tenacibaculum* genus has several species which are pathogen to fish [68–70]. The bacterial community of culture water had a great difference from that of bioreactors. And the function of most bacteria in culture water is not sure.

### Probiotics and potential pathogen

The growth of the aquaculture industry is hampered by unpredictable mortalities, many of which are caused by pathogenic microorganisms. Bacterial diseases have been attributed to biological production bottlenecks in intensive aquaculture [71]. Besides a microbial community that purifies the water, microbiota in RAS can also harbor pathogens or produce off-flavor-causing compounds [72]. Because of a low dilution rate and high organic loading, pathogens might accumulate more in RAS biofilters than in single-pass systems [73]. Besides *Vibrio* as a traditional pathogen, *Streptococcus* sp. [74], *Nautella italica*, and *Pseudoalteromonas piscicida* [54] also have threats to shrimp. Frequent application of antibiotics might lead to antibiotic resistance and destroy bacterial community structure. The use of antimicrobials to control diseases should be limited in RAS, and more attention should be given to alternative approaches [75, 76]. Probiotics is a safer and more effective method to manage bacteria and control pathogenic bacteria via competitive exclusion, producing a specific inhibitory substance, competition for nutrients, producing antagonist for quorum sensing mechanism and improving immunity [71]. The most commonly used probiotic species include *Lactobacillus*, *Bacillus*, *Pseudomonas*, nitrobacteria, etc. In addition, ‘neutral bacteria’ could also contribute to the stabilization of the microbial community and even play a primary role in biosecurity [77]. According to the result of bacteria composition, the proportion of probiotics was relatively low. And potential pathogen, especially *Nautella* in culture water, had a high frequency. Though the function of most bacteria in the systems is not sure, ‘neutral bacteria’ may take great contribution to the balance of potential pathogens and candidate probiotics.

### The effects of water recirculation on RAS

Increasing the water recirculation ratio can increase the number of times water flows through the water treatment process, increase the hydraulic load, and improve the removal efficiency of solid particles and ammonia. Some studies have reported that shorter HRT is conducive to the purification of water quality [24, 78, 79]. Increasing the water recirculation ratio will increase the turbulence of the water. And the nitrification rate could be significantly improved by increasing the turbulence [80].

The effect of water recirculation ratio on ammonia removal is to change the number of times water flows through the bioreactor, on the other hand, to change the structure of the microbial community in the bioreactor. Water recirculation ratio was an important factor affecting microbial community structure [14, 17, 21, 79]. The proportion of dominant groups showed a trend with the variety of water recirculation ratio in bioreactor and culture water. In the middle period of cultivation, the dominant family of bioreactor was *Rhodobacteraceae*, accounting for 30.2–49.6% of the community, which increased with the increase of water recirculation ratio (Fig. 2 a). At the end of cultivation, the dominant family of bioreactor was *Phytophthoraceae*, accounting for 16.0–30.1% of the community, which decreased with the increase of water recirculation ratio (Fig. 2 b). In the culture water, the dominant phylum was *Proteobacteria*, accounting for 77.9–96.4%, which increased with the increase of water recirculation ratio (Fig. 4 a). And the dominant families and genus of culture water showed different trends with the variety of water recirculation ratio (Fig. 4 b, c). The microbial community of RASs was significantly changed by water recirculation ratio, which could be furtherly confirmed by UPGMA tree. UPGMA tree (Fig. 6) reflected the similarity of different samples. In bioreactors, LC and MC had more similarity, however in culture water, HC and MC had more similarity. In the bioreactor, increasing water recirculation ratio will increase the shear force on the surface of biofilm. Bacteria with strong adhesion ability can grow on the surface of the substrate in the bioreactor. In addition, the flow rate on the surface of biofilm also affects the absorption and utilization of nutrients by microorganisms in water. For microorganisms in culture water, water recirculation ratio will affect the number of times water passes through UV, resulting in the bacteria with stronger resistance to UV or more reproductive have more advantages.

Water recirculation ratio not only affects the dominant groups of microbial communities but also affects some functional microbial groups. Although there are only three kinds of nitrifying bacteria in the bioreactor, *Nitrococcus*, *Nitrosomonas* and *Nitrospira*, and their proportion in the microbial community was not significantly different among LC, MC and HC, the proportion of the three nitrifying bacteria in the community increased with the increasing of water recirculation ratio, which was (0.6 ± 0.5) %, (0.9 ± 0.4) % and (1.1 ± 0.6) %, respectively. That is, the number of nitrifying bacteria was more under the high water recirculation ratio. For the *Vibrio* in bioreactor, its proportions in LC, MC and HC were (3.2 ± 0.5) %, (1.4 ± 0.1) % and (0.5 ± 0.0) %, respectively. The proportions of *Vibrio* in bioreactor were decreased with the increases in water recirculation rate (*P* < 0.05). For potential pathogen *Nautella* (Fig. 4 c), the proportion in the water of LC was significantly more than that in MC and HC (*P* < 0.05). Increasing water recirculation ratio is beneficial to reducing the number of potential pathogens in bioreactors and aquaculture waters. The water recirculation ratio had a great effect on bacterial community structure, which could be a useful method to manage RAS.

**Fig. 9:**
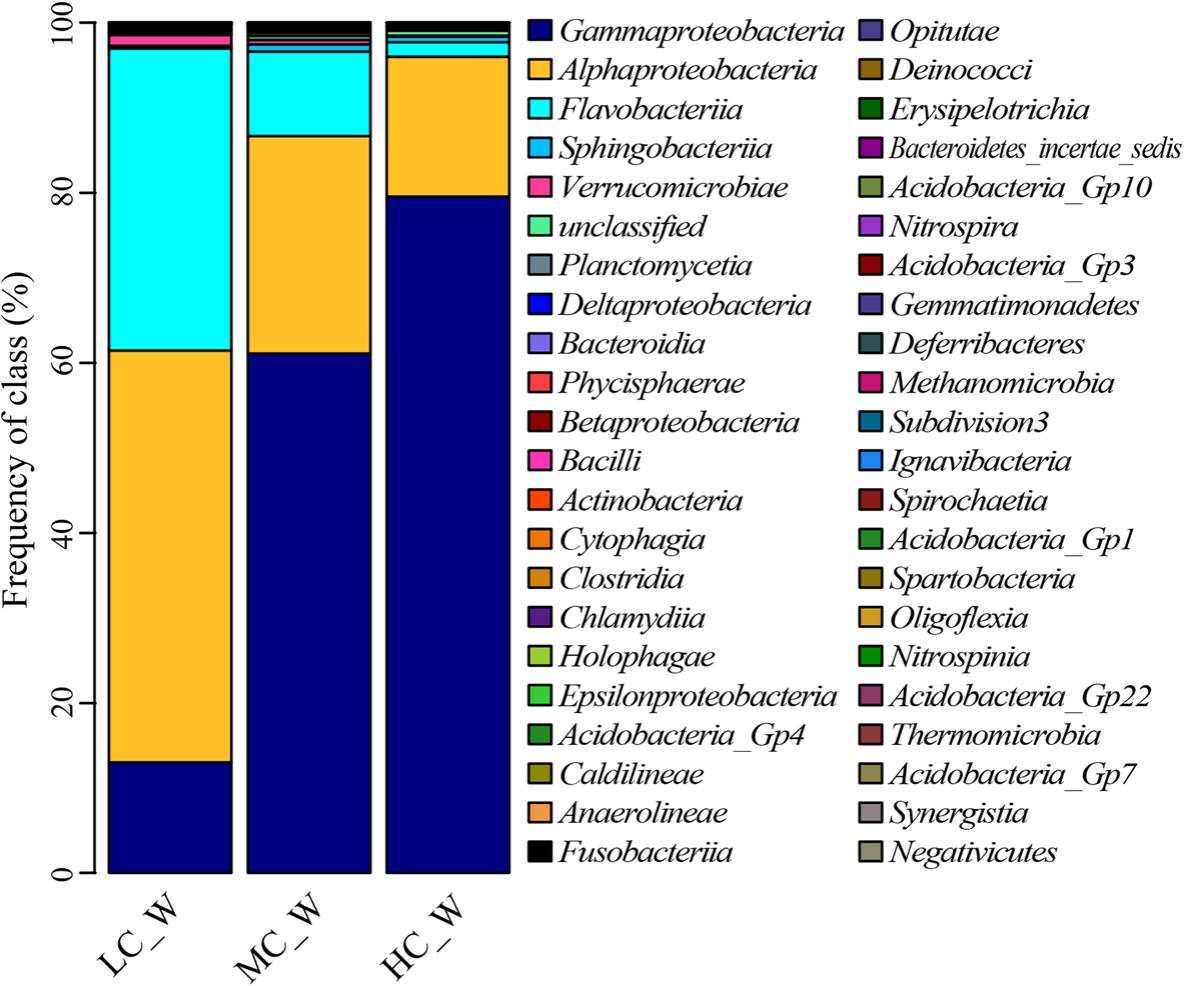
Taxonomic assignment of bacteria in culture water at class level shown as a percentage of the total microbiota

Increasing water recirculation rate could improve water quality, reduce the number of pathogenic bacteria and promote shrimp growth. The proportion of potential pathogens *Nautella* and *Tenacibaculum* in culture water of LC treatment were higher than that in MC and HC, which assumed to be the reason for lower SGR of shrimp in LC. The mean weight of shrimp in LC was significantly less than that in MC and HC, but the survival, yield and FCR of LC were higher than MC and HC. In view of the significantly different in the bacterial community among LC, MC and HC, there was an important relationship between shrimp culture and the bacterial community. The higher mean weight of shrimp in MC and HC was partly due to the higher mortality. On the other hand, a higher proportion of *Pseudoalteromonas* in MC and HC may promote the growth of shrimp. The higher mortality of shrimps in MC and HC maybe due to *Aestuariibacter* and *Thalassotalea* in view of their dominant status in the bacterial community of culture water, meanwhile their proportions were much higher than that in LC. *Aestuariibacter* and *Thalassotalea* belong to the *Gammaproteobacteria* class. At the class level, the proportions of *Gammaproteobacteria* in the bacterial community of culture water were 13.0, 61.1 and 79.6% in LC, MC and HC, respectively (Fig. 9). The high proportion of *Gammaproteobacteria* in culture water may be the reason for decreasing the survival rate of *L. vannamei*. In view of the unclear function of most bacteria in water, the relationship between bacteria in culture water and *L. vannamei* needs further study.

## Conclusions

In the microbial community of RAS for *L. vannamei*, the most dominant phylum is *Proteobacteria*, followed by *Planctomycetes*, *Bacteroidetes*, *Actinobacteria* and *Verrucomicrobia* phylum. *Rhodobacteraceae* and *Planctomycetaceae* are the dominant families in the biofilter, while culture water community is dominated by *Proteobacteria* including the dominant families of *Rhodobacteraceae*, *Aestuariibacter*, *Thalassotalea*, and *Pseudoalteromonas*. Compared with culture water, bioreactor has a higher abundance and diversity of the microbial community and plays a more important role in the environmental regulation of RAS. Although several studies have been investigating the culture system microflora, the knowledge regarding the roles of environmental microbiota in RAS remains limited. The function of most dominant genera in RAS are unknowns and need further research.

Water recirculation rate as an important parameter of RAS affects the bacterial community of bioreactor and culture water. The proportions of dominant groups show a trend with the variety of water recirculation rate in bioreactor and culture water. The proportions of some microbial groups, such as nitrifying bacteria, *Vibrio* and *Nautella*, in the microbial community is also affected by the water recirculation rate. Water quality indexes such as ammonia, nitrite and turbidity decrease with the increasing of water recirculation rate. Though the growth rate and final weight of shrimp were the highest under medium water recirculation rate, the survival and yield have an inverse correlation with water recirculation rate. The higher mortality rate of shrimp under medium and high water recirculation rate assumed to be caused by the excessive *Gammaproteobacteria* in the bacterial community. The increasing water recirculation rate could change microbial community constitution, improve water quality, and promote shrimp growth, however, *L. vannamei* has better performance of survival and yield under low water recirculation rate. Water recirculation rate is an effective method to manage RAS of *L. vannamei*, and the impacts of water recirculation on RAS needs further study, especially in the application of low level of water recirculation.

## Methods

### Experimental systems

**Fig. 10:**
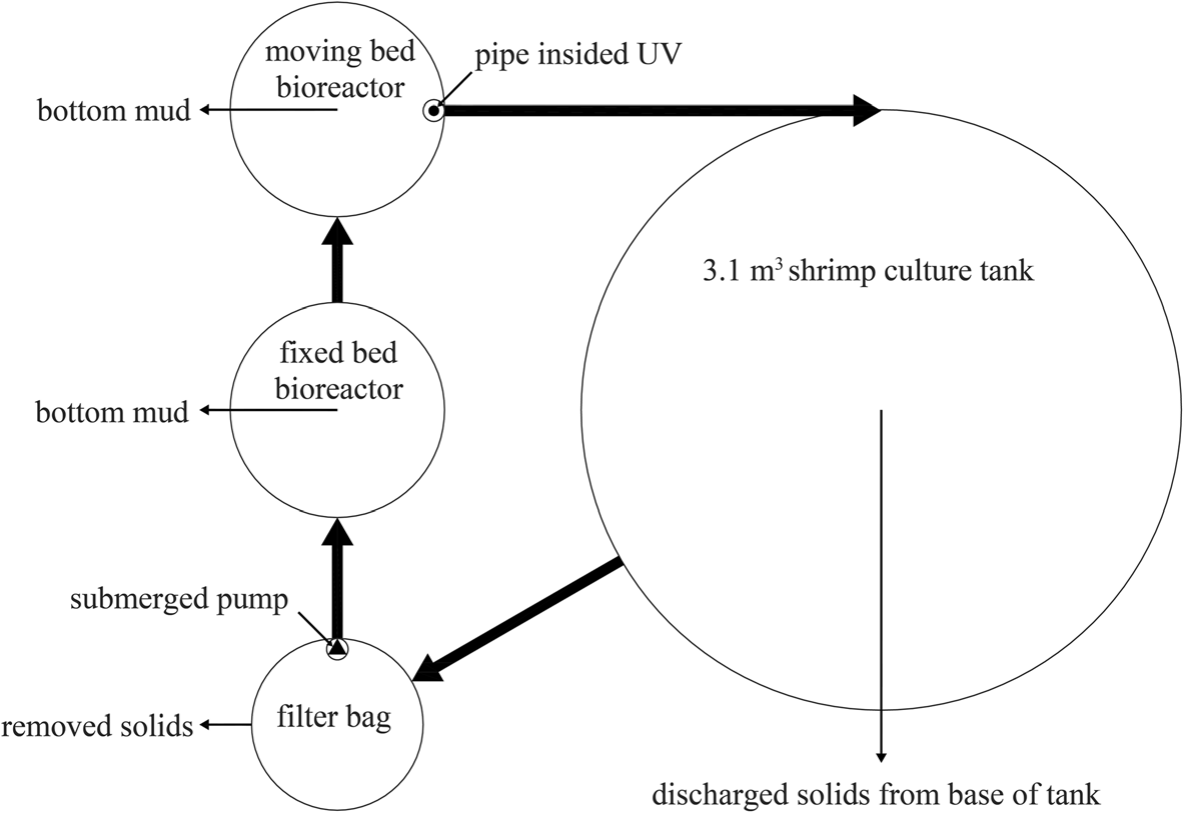
Summary diagram of an individual experimental recirculating aquaculture system employed in this study

The experiment lasted 91 days from June 2nd to September 1st. RASs was indoor and adopted natural lighting trough light-transmitting plate. Each RAS consisted of one circular fiberglass culture tank (3.1m^3^) and one filter bag with 75 μm pore size for particle removal, followed by a fixed bed bioreactor (FBBR, 0.6 m^3^) and a moving bed bioreactor (MBBR, 0.6 m^3^) for removal of toxic ammonia and nitrite, after which the water was disinfected by UV and returned to the culture tank (Fig. 10). The stock density is 1000 shrimp/tank (322ind/m^3^). No water exchanged in the first 2 weeks; then water was exchanged twice a week with the water exchange rate 14–28%.

### Experimental design

Three water flow rates were set for the experiment with the water recirculation rate of 5, 10 and 15 day^− 1^, representing low (LC), medium (MC) and high (HC) recirculation rate respectively. Each recirculation rate had two RASs. Shrimps were fed with formulated feed (42% crude protein) five times a day. The feeding amount of each tank was the same. The feeding amount was 4% of total shrimp weight at the initial stage, and gradually increased with the growth of shrimps. Water samples were collected from the culture tank every week for the measurement of water quality to reflect the water environment [81]. One bacteria samples were collected from each bioreactor for the measurement of the microbial community at the medium-term and end. Besides, one bacteria sample in culture water was also collected via 0.22-μm-pore-diameter filter membrane at the end of culture from each system. For bacterial samples, one sample was taken from each RAS, i.e. two parallel bacterial samples were taken from each water recirculation ratio. Bacteria samples were stored at − 80 °C before usage. At the end of culture, 20 shrimps were collected from each system for the measurement of individual weight.

### Measurements

Shrimp weight was measured by electronic scales (Mettler Toledo, Shanghai, China). DO, pH, temperature and salinity were recorded with YSI (YSI Incorporated, Yellow Springs, OH, USA). Turbidity was measured by turbidimeter (Yuefeng, Shanghai, China). Total ammonia was measured by phenate method [81], nitrite was measured by N-(1-naphthyl)-1, 2-diaminoethane dihydrochloride spectrophotometry [82], Vibrio number was enumerated by TCBS agar media [83–85] for reflecting the status of pathogenic microbes. The growth performance of shrimp was reflected by initial weight, final weight and specific growth rate (SGR = 100 × (Ln (final weight) – Ln (initial weight)) / culture days).

Total DNA of bacterial samples was extracted using the TIANamp Bacteria DNA Kit (Tiangen Biotech, Beijing, China). The DNA concentration was determined using NanoDrop Spectrophotometer (Thermo Scientific, USA), and DNA integrity was confirmed by agarose gel electrophoresis. Microbial DNA was amplified by polymerase chain reaction for the V3–V4 region of the 16srRNA gene. After purification, samples were mixed in equal concentrations and sequenced by Illumina-MiSeq. The raw reads were deposited into the NCBI Sequence Read Archive (SRA) database (Accession Number: SRP214732).

### Statistical analysis

The statistical analysis was performed using SPSS program version 13.0 (SPSS, Chicago, IL, USA), an independent samples T-test was conducted to compare the significant differences among the systems with different water recirculation rates on water quality and shrimp growth, one-way ANOVA was conducted on the change of water recirculation rates among the systems, and Pearson correlation analysis and linear regression analysis were conducted to reveal the relationship among shrimp growth, water recirculation rate and water quality indexes.

These sequences of bacteria samples were clustered using Usearch into operational taxonomic units (OTUs), based on 97% sequence similarity. The sequences were aligned against the bacterial NCBI database for taxonomic classification. Alpha-diversity indices (Chao index and Simpson index) for each sample were calculated using R [86]. The Chao1 index quantifying species richness was calculated using the formula:

$$ {S}_{\mathrm{Chao}1}={S}_{\mathrm{obs}}+{n}_1\ \left({n}_1-1\right)/\left(2\times \left({n}_2+1\right)\right) $$

Where *S*_obs_ is the observed number of OTUs, *n*_1_ is the number of OTUs with only one sequence, and *n*_2_ is the number of OTUs with only two sequences [87]. The Simpson index quantifying community diversity was determined as:

$$ {D}_{\mathrm{Simpson}}=\left({\sum}_{i=1}^{S_{obs}} ni\left( ni-1\right)\right)/\left(N\left(N-1\right)\right). $$

Where *S*_obs_ is the number of observed OTUs, *n*_i_ is the number of individuals in the *i*th OTU, and *N* is the total number of individuals in the community [88]. Based on the unweighted UniFrac distance metric [89] estimated by QIIME [90], principal co-ordinate analysis (PCoA) and cluster analysis (CA) was conducted using the unweighted pair group method with arithmetic mean (UPGMAM) method to estimate β-diversity and visualize microbial community diversity among samples [91]

## Data Availability

All supporting data generated during this study are included in this published article.
